# Aesthetic Chills: Knowledge-Acquisition, Meaning-Making, and Aesthetic Emotions

**DOI:** 10.3389/fpsyg.2016.01093

**Published:** 2016-08-04

**Authors:** Felix Schoeller, Leonid Perlovsky

**Affiliations:** ^1^Department of Media, Cognition and Communication, University of CopenhagenCopenhagen, Denmark; ^2^Psychology, Northeastern UniversityBoston, MA, USA

**Keywords:** knowledge instinct, meaning, knowledge, pleasure, memory, curiosity, motivation, aesthetic chills

## Abstract

This article addresses the relation between aesthetic emotions, knowledge-acquisition, and meaning-making. We briefly review theoretical foundations and present experimental data related to aesthetic chills. These results suggest that aesthetic chills are inhibited by exposing the subject to an incoherent prime prior to the chill-eliciting stimulation and that a meaningful prime makes the aesthetic experience more pleasurable than a neutral or an incoherent one. Aesthetic chills induced by narrative structures seem to be related to the pinnacle of the story, to have a significant calming effect and subjects describe a strong empathy for the characters. We discuss the relation between meaning-making and aesthetic emotions at the psychological, physiological, narratological, and mathematical levels and propose a series of hypotheses to be tested in future research.

## Introduction

Aesthetic chills seem to be a universal emotion (McCrae, 2007). The phenomenon invokes a wide variety of issues of interest to psychologists, such as the function of music for the cognitive system (Blood and Zatorre, 2001; Harrison and Loui, 2014; Perlovsky, 2015b), the relation between cognition, social recognition and empathy (Keltner and Haidt, 2003), intelligence and collective intelligence (Sully, 1892; Algoe and Haidt, 2009), fear and expectations (Maruskin et al., 2012; Schoeller and Perlovsky, 2015), aesthetic emotions and natural curiosity (Perlovsky, 2001; Schoeller, 2015a), aesthetic emotions and the drive for knowledge and meaning (Perlovsky, 2006; Chater and Loewenstein, 2015) and, at a more general level, the function of artistic, scientific, and religious behaviors in human societies (Schoeller, 2015b). In order to understand, describe, and predict aesthetic emotions, one needs not only to assess what can elicit them but also what can suppress them. Thus, a scientific study of emotions of the beautiful is not only concerned with how they might emerge (elicitors) but also by the conditions that might prevent them (inhibitors). To our knowledge, that latter dimension of the problem has not been empirically studied before at the psychological level (but see Goldstein, 1980). To clarify this issue, we decided to focus on the problem of aesthetic chills (hedonic non-thermoregulatory shivering in humans). In this introductory section we briefly introduce a mathematical model relating aesthetic emotions to knowledge-acquisition (Perlovsky, 2006, 2014a). This model extends Kantian intuitions (Kant, 1790/1987, Introduction VII, 191, §6) and has predicted a number of the mechanisms of the mind. Several of these predictions have been experimentally confirmed (Binder et al., 2005; Bar et al., 2006; Masataka and Perlovsky, 2012a,b, 2013; Perlovsky et al., 2013; etc.). In order for them to be replicated in a different socio-cultural setting, we report a series of experiments tentatively confirming the predictions relating emotions of the beautiful to the process of meaning-making in humans. In this introductory section, we discuss the methodological considerations that have led to our experimental study and review the relevant data pertaining to the phenomenon under consideration.

### The instinct of knowledge

In 1987, Grossberg and Levine published a seminal article presenting a theory of drives and emotions where drives are modeled as internal sensors measuring vital bodily parameters and indicating their safe ranges (Grossberg and Levine, 1987). When a parameter is outside its safe range, the information is transmitted by neural signals to decision-making parts of the brain initiating appropriate decisions and behavior. These neural signals are experienced internally as emotions motivating behavior. A simple example is that of blood glucose level, when the sugar concentration is below a certain level, it is felt as hunger and the subject devotes more attention to finding food. This theory has been extended from bodily needs to learning (Perlovsky, 2001, 2006; Perlovsky et al., 2011). In humans and higher mammals, survival and satisfaction of bodily needs require a correct understanding of the surrounding world. An essential aspect of perception and understanding consists in matching external sensory signals to mental representations, that is, to memories (Kosslyn, 1994; Grossberg, 2014). A new object is never exactly the same as memories of similar objects and matching mental representations to sensory signals requires modifying existing representations. Therefore, humans possess a drive to modify existing representations to match patterns in sensory signals. This improvement of representations (and creation of new ones) constitutes knowledge-acquisition; it is fundamental for survival and therefore driven by a primary drive called the knowledge instinct, KI. Thus, learning consists in improving mental representations for better correspondence to the world. A mathematical model of KI has been discussed in the above references; knowledge is modeled as a similarity between mental representations, *M*(*m*), and patterns in sensory data, *X*(*n*):

(1) $$\begin{array}{l} L=\left\{\underset{n}{\prod }\left[\underset{m}{\sum }l\left(X\left(n\right)|M\left(m\right)\right)\right]\right\} \end{array}$$

Here, *l* is a conditional similarity of the data given the representations. Sum (Σ) over *m* accounts for all the representations, and product (∏) over *n* accounts for all the data available to sensors. In this way, similarity *L* accounts for all perceptions, it measures the total similarity between all sensory data and all mental representations, and models the entire knowledge perceived in the world. This model does not represent all the intricacies of actual neural mechanisms of perception (for example see publications of Grossberg and colleagues, and references therein), but it is sufficient for our purposes of modeling conceptual-emotional structure of the brain-mind at higher levels. Increase or decrease of this similarity measure models satisfaction-dissatisfaction of KI.

Like all satisfactions-dissatisfactions of drives, changes in (1) model emotions. Since these emotions are related to knowledge, they are aesthetic emotions (Kant, 1790/1987; Perlovsky, 2014a). As mentioned, the neural mechanisms of perception of food and other objects involve matching mental representations of objects to patterns in sensor signals (Kosslyn, 1994). Mental representations are organized into an approximate hierarchy from perceptual elements, to objects, to contexts and situations, and higher up to abstract concepts (Simon, 1967; Perlovsky, 2001). The evolutionary purpose of evolving the hierarchy is to enable the formation of abstract concepts. For example, the representation “symphony hall” unifies lower-level representations of objects (rows of chairs, listeners, scene, performers, etc.) into a unified concept of the symphony hall. Similarly, concepts of a symphony hall, concerts, (etc.) are unified into a concept of “conservatory,” and “higher up” the hierarchy to “culture,” etc. The hierarchy of the human mind above objects, to contexts, situations, and abstract concepts is modeled by extending similarity (1) combining cognition and language to a hierarchical model (Perlovsky, 2004, 2009a, 2013a; Perlovsky and Ilin, 2010, 2012).

(2) $$\begin{array}{l} L_{hierarchical}=\prod L\left(h\right) \end{array}$$

Here, L(h) is a similarity of the type (1) at level *h* in the hierarchy and product is over all hierarchical levels *h*. At the very “bottom” level *h* = 1 data *X* are sensory or motor signals; at every next level *h, X* are the bottom-up signals, coming from the lower level (*h*–1) and representations, *M*, are the top-down signals.

### Aesthetic emotions

Aesthetic emotions as discussed above are related to the knowledge instinct. In this way, they are similar to all emotions which according to the Grossberg-Levine theory (Grossberg and Levine, 1987) correspond to a satisfaction or dissatisfaction of a given instinct. A mathematical model of KI is given by Equation (1), correspondingly changes in (1) model aesthetic emotions (Perlovsky, 2001, 2006, 2010; Perlovsky et al., 2010). Change in KI, for example during visual perception, involves a change in representations *M* stimulated by a pattern *X* in the observer's visual field. Somewhat simplifying, this process can be described as follows, a representation most likely to be similar to *X* is modified to fit *X*. When a fit occurs, *M* ~ *X*, the mind recognizes the object *X*. Mathematically, it is modeled by a local maximum of KI. Adequacy of this mathematical description (along with mathematical details described in the references) has been experimentally demonstrated in Bar et al. (2006). Coherent with a long philosophical tradition, motivations for these fitting processes are referred to as *aesthetic emotions*. We do not consciously experience such emotions during the perception of everyday objects, we do not feel aesthetically elated when recognizing, say, a chair. Yet, when we do not recognize objects around us or their properties, we may feel disturbed or even scared. This is a staple of thrillers: when a situation is not recognizable, one may experience horror chills (*frissons d'effroi*). Mathematically, it is modeled by a local minimum of KI, in our memory there is no representation *M* that could be fit to perceived patterns *X* around us. To our knowledge, this is the only mathematical model of aesthetic emotions in the literature.

**Figure 1:**
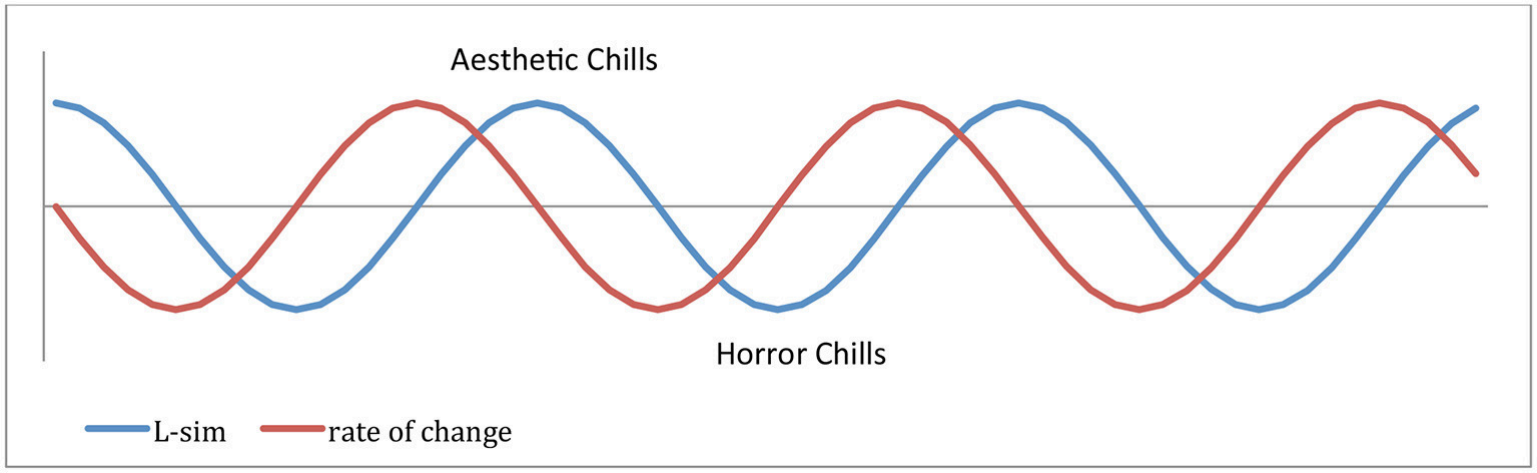
**Notional illustration of KI changes**. Satisfaction of KI, aesthetic chills, corresponds to max *L*, and *L*′ = 0; dissatisfaction of KI, horror chills, corresponds to min *L*, and *L*′ = 0. Vertical axis is Similarity *L* and horizontal axis is Time in arbitrary units.

The topic of this paper are positive aesthetic chills, corresponding to a maximum of KI, when our mind can find representations *M* matching objects in environmental conditions, *M* ~ *X*. Neural mechanisms of representations *M* near the bottom of the mental hierarchy, say at the level of objects, evolved in genetic and cultural evolution and in individual learning for the purpose of object recognition. These representations unify into objects individual pixels projected from the retina to the primary visual cortex (a mechanism similar in both humans and higher animals). Representations of abstract ideas at higher levels evolved for unifying lower level representations into higher level ideas. These mechanisms require language and are uniquely human (Perlovsky, 2011, 2013a). When understanding ideas of importance higher in the hierarchy, we may experience aesthetic emotions consciously. If we continue these arguments toward the top of the hierarchy, we can understand the contents of the top representation: it attempts to unify all representations below, the entire life experience (Perlovsky, 2009a, 2014a). Above, we described the top of the hierarchy as a single representation. Actually the theory in given references predicts that representations near the top are vague and mostly unconscious. We may discuss these representations and the idea of the top of the hierarchy with the help of language. Ideas of the beautiful are discussed in many literary work and this is rendered possible because, parallel to a cognitive hierarchy, the mind-brain also develops a language hierarchy, which is crisp and conscious throughout the hierarchy. Learning language description of the top contents may improve understanding of the cognitive contents by connecting language to one's life experience. In this paper, we make a step toward experimental verification of the theoretical predictions about aesthetic emotions near the top of the hierarchy. The processes of changing KI, its increase in the process of understanding and its decrease when encountering new situations are continuously going on at multiple hierarchical levels. A simplified illustration of this process at a single level is shown in Figure 1 (KI is shown in blue). The change in KI, as discussed, models aesthetic emotions (shown in red). At lower hierarchical levels these emotions could function below the threshold of consciousness. Near the top of the hierarchy they motivate the understanding of the highest meaning and may be experienced as emotions of the sublime or aesthetic chills. The strongest emotions are experienced near the peak of the curve, when changes slow down. In a later section we shall compare this description to the subjective reports of the participants in our experiments.

### Methodological considerations

This study pertains to aesthetic chills and their relations to the knowledge instinct. As an early study in this wide field of research, it is by necessity explorative in nature. We aim at determining a set of elicitors and inhibitors in order to improve our understanding of the mechanism of aesthetic emotions and verify a set of theoretical predictions. If we succeed at eliciting chills and if we succeed at inhibiting them, then we may study their causes and effects in the laboratory. Our general hypothesis, suggested by the mathematical model introduced above, is that aesthetic emotions correspond to satisfaction or dissatisfaction of the knowledge instinct. This theory of aesthetic emotions is an extension of Grossberg and Levine (1987) theory of drives and emotions and thus our experimental procedure may be understood in analogy to any other vital need such as hunger or reproduction.

One particular aspect of a vital need is its *dynamic*, the variation of its intensity over time. Exposing healthy subjects to pornographic material should induce sexual excitement, exposing them to appetizing food should induce hunger. This kind of pre-activation of the corresponding system is called *priming*, a standard procedure in experimental psychology. At a general level, priming can be understood as a bottom-up activation by prior inputs which leaves a residual activity trace and makes it easier for the primed cells to be activated by similar future inputs (Grossberg, 1982a; Grossberg and Stone, 1986; Levine and Leven, 1992). For example, a hunger drive representation (a neural subsystem simultaneously coding the level of a drive and the possibility of satisfying it) is highly active whenever the organism is hungry and when there are available food or cues signifying the future availability of food (Grossberg, 1971, 1982b). We extended the method of priming to the field of cognition. In our experimentation, prime 1 should arouse the subject (pornographic material, succulent meal), prime 2 should not have any such effect (neutral material), and prime 3 should have the opposite effect (repulsive material).

To test the working hypothesis, we chose to use primes that are syntactically regular sentences but which meaning vary greatly in relation to the aim of our study—the meaning near the top of the cognitive hierarchy. The first prime (the meaningful prime) should require thoughtful cognition, the second prime (the neutral prime) should not require any cognitive effort, and the third prime (the incoherent prime) should be a sentence devoid of meaning. For this purpose, after a preliminary study conducted on a similar population, we chose to use (i) a sentence from the French philosopher Pascal (“the supreme function of reason is to show man that some things are beyond reason”), (ii) a sentence from a children's book (“I once saw an apple in a tree”), and (iii) a classic example from Noam Chomsky illustrating that syntax might be independent from semantics (“colorless green ideas sleep furiously”). If you ignored its semantic content, the Chomsky prime would appear like an ordinary and simple sentence, as its syntactic structure is unexceptional. The particularity of this sentence is that its words violate *semantic selections* (Chomsky, 1957). Things should not be both colorless and green (as green is a color), ideas should not have color (as they are nonphysical), ideas cannot sleep (as they are inanimate), and sleeping cannot be furious (as it is non-intentional). The Pascal prime is also syntactically regular, though it has a complex signification. This prime describes a wide range of reality and, *ceteris paribus*, it is more likely than the neutral sentence to engage representations near the top of the hierarchy (this was confirmed by the participants verbal reports). Its important property as a sentence is that the key word “reason” is syntactically buried into its constituent parts. This makes disentangling the meaning—understanding why reason is used twice—requires a cognitive effort but, contrary to the Chomsky sentence, it can be achieved (i.e., it offers a solvable problem) and the deep meaning of this sentence is accessible to consciousness.

### Aesthetic chills

Among aesthetic emotions, the case of aesthetic chills (non-thermoregulatory hedonic shivering) seems to be an ideal candidate of study, mainly because they appear to be universally valid quantifiable phenomena (McCrae, 2007; Benedek and Kaernbach, 2011). When considering non-thermoregulatory shivering, the first noticeable thing is that there seems to be two contradictory types of chills, chills elicited by the subject's greatest fear and chills elicited by the subject's greatest hope (for further discussion refer to Maruskin et al., 2012). Chills are also known to be elicited by tonal music of both positive and negative valence (Goldstein, 1980; Panksepp, 1995). This has led us to the hypothesis that chills might correspond to an event where KI-similarity reaches a local maximum or minimum value and the rate of change of the similarity function tends toward a null value (Figure 1). This makes sense both psychologically (compare Halpern et al., 1986 to Sloboda, 1991) and physiologically (compare Blood and Zatorre, 2001 to Zald and Pardo, 2002). Aesthetic chills may be elicited in artistic, scientific, and religious contexts (Schoeller, 2015b). However, they have mainly been studied in the laboratory using tonal music as an elicitor (Sloboda, 1991; Harrison and Loui, 2014). Musical chills are known to provoke changes in autonomic and other psycho-physiological activity (Blood and Zatorre, 2001). Measurements of regional cerebral blood flow (rCBF) identified changes in structures associated with the brain reward circuitry (Berridge and Kringelbach, 2008). Regression analysis revealed rCBF increases in left ventral striatum, dorsomedial midbrain, and rCBF decreases in right amygdala, left hippocampus/amygdala, and ventral medial prefrontal cortex (Blood and Zatorre, 2001). The pattern of activity observed in correlation with music-induced chills is similar to that observed in other studies of highly pleasurable emotions (e.g., Ketter et al., 1996). A neural theory of how pleasure might be related to knowledge-acquisition and understanding identified a similar network involving association cortex, dorsolateral prefrontal cortex, orbitofrontal cortex, striatum, opioids, and dopamine (e.g., Levine, 2012). Candidate neural mechanisms are discussed in Levine and Perlovsky (2008), Levine (2012), Perlovsky and Levine (2012). Further evidence for our hypothesis that aesthetic emotions correspond to maxima of KI-similarity is provided by pharmacological studies on the role of endogenous opioids in learning, retention, and memory. Studies in animal pharmacology suggest that the opioid receptor antagonist naloxone (C_19_H_21_NO_4_; *M* ≈ 327.37 Da) tends to *impair* retention and learning performances in rats (Saha et al., 1991). An exploratory study conducted by Goldstein demonstrated that blocking opioid receptors with the same synthetic molecule significantly decreased or inhibited chill responses (Goldstein, 1980). However, no *psychological* study has yet analyzed the relation between aesthetic chills and knowledge-acquisition. We thus proceed to investigate it in a series of experiments.

To summarize this introductory section, let us formulate the following hypotheses which tests are reported in this paper: (i) aesthetic chills correspond to an event where KI-similarity between bottom-up sensory signals and top-down mental models reaches a positive maximum whereas horror chills correspond to an event where KI-similarity reaches a local minimum; (ii) when KI-similarity reaches a positive maximum near the top of the mental hierarchy humans experience aesthetic emotions, which contents are elucidated in this paper; (iii) a coherent prime should increase the likelihood of subjects experiencing chills, whereas an incoherent prime should decrease it.

## Methods

In a series of quantitative and qualitative studies, we tested experimentally the relation between aesthetic emotion and meaning-making using the methodology of priming. We inquired into the phenomenology of the aesthetic experience by asking our subjects what it felt like to experience chills and we also studied the properties of chill-eliciting narratives by asking our subjects after the experiment for a description of a scene in a narrative (film, book, play, etc.) that gave them the chills or made them shivers.

### Quantitative study (priming and KI)

To test our hypotheses, we used the methodology of semantic priming. Priming is a bottom-up activation by prior inputs that leaves a residual activity trace that makes it easier for the primed cells to respond to similar future inputs. In order to engage our subjects in epistemic behavior, we followed Berlyne's intuition and introduced the semantic cue with a question (“please concentrate, now what do you think about this sentence?”) (for discussion see Berlyne, 1960, p. 289, see also Inan, 2012). We divided our subjects into three groups, each primed with a different text. Our treatment group (GROUP 1) is exposed to a coherent existential prime supposed to arouse their knowledge instinct by activating without too much resistance the categories at the top of the mental hierarchy (the models which encompass the widest range of reality and that are consequently highly difficult to manipulate by the subject). We chose for this purpose a quote from the philosopher Pascal: “the supreme function of reason is to show man that some things are beyond reason.” Post-experimental interviews with subjects positively revealed that the prime was successful in engaging epistemic behavior and subjects in this priming group successfully experienced chills. A second group of subjects (GROUP 2) were exposed to a neutral prime (“I once saw an apple in a tree”) and we exposed the third group of subjects (GROUP 3) to an incoherent prime (Chomsky's famous example “colorless green ideas sleep furiously”). Post-experimental interviews with subjects positively revealed that the prime was indeed rated by participants as “incomprehensible,” “incoherent,” “paradoxical,” etc. Finally, we also decided to quantify the amount of pleasure brought by the experience to the subject. This was done by using an analog rating scale (0 to 10).

#### Materials

Audiovisual stimuli were presented using a computer screen (Philips Gioco3D, 238G4; 27″/68.6 cm) and two Dell speakers at a fixed sound volume. No study has yet analyzed chills as elicited by audio-visual stimuli and very little information is available regarding narrative chills. After a preliminary study using scenes obtained through responses to a survey inquiring into the properties of chill-eliciting situations, we chose for the stimulation a film involving a dance sequence choreographed by Angelin Preljocaj and the adagio from Mozart's 23rd piano concerto, K488 (easily available online using the keywords, “Air France,” “l'envol”). This stimulation is 60 s long and successfully elicited aesthetic chills in our population. For further discussion on audiovisual contents more likely than others to elicit chills refer to Schoeller (2015b). For evaluation and presentation of data, we used SPSS and Matlab.

#### Participants

The experiment was conducted in Denmark at a laboratory of the University of Copenhagen. A total of 30 international students at the University of Copenhagen from the Media, Cognition and Communication department participated in the experiment (16 females, 14 males; age range: 20–33). On their arrival at the laboratory waiting room the participants were randomly assigned to one of the three priming conditions.

#### Procedure

The participant entered the laboratory, sat in front of a blank screen in a comfortable chair. She was provided with a factual account of what is going to happen next and gave her informed consent. Two electrodes were wrapped around the middle phalanges of her non-dominant index and middle finger. The experimenter then proceeded to launch the experiment and left the room. For 90 s the subject was asked to stay still while shown a neutral picture of the ocean. This was done in order to obtain a baseline response for the statistical analysis. The subject was then asked to concentrate and is exposed to a prime that she had been randomly assigned to at arrival in the laboratory. The exact wording of the prime was as follows: “Please concentrate. Now, what do you think about this sentence? (priming).” After 90 s of prime, the subject was exposed to the chill-eliciting stimulus (a film excerpt). The stimulation was 60 s long. The subject was then exposed again (for 90 s) to the picture of the ocean in order to perfect our baseline. Once the experiment was finished, the experimenter came back in the room, disconnects the sensors and provides the subject with a questionnaire. The questionnaire contains (i) Kashdan and Fincham's curiosity measure, (ii) demographics, (iii) an analog rating scale for the amount of pleasure (0–10) felt by the subject during the film (iv) narratological questions regarding chill-eliciting scenes in narratives, and (v) various open phenomenological questions (such as “please describe in your own words what you have felt during the film that you have just seen,” etc.). The subject was asked if she was familiar with any of the main properties of the stimulus (music, location, choreographer, familiarity to ballet in general) and if she had any idea about the aims of the study. Finally, the subjects were thanked for their participation and fully debriefed. Each session lasted about 10 min.

#### Ethics

The experiment is in compliance with the Helsinki Declaration. All participants gave their voluntary informed consent and we followed the Ethics Code of the American Psychological Association. All participants were informed about the purpose of the research, about their right to decline to participate and to withdraw from the experiment and about the limits of confidentiality. We also provided them with a contact for any questions concerning the research and with the opportunity to ask any questions regarding the phenomenon under study (aesthetic chills) and receive appropriate answers. All participants reacted positively to the experiment and were thankful for the opportunity to learn about the phenomenon.

### Qualitative study (narratology and phenomenology)

A qualitative data analysis was used to study two aspects of the problem: the phenomenology of the experience and the narratology of chill-eliciting scenes. The aesthetic experience, in addition to being related to knowledge-acquisition, involves two components, a hedonistic dimension and an attentional component (Schaeffer, 2015). Indeed, one and the other are strongly related since emotions have attentional properties (Simon, 1967). We placed the emphasis here on the *hedonistic* dimension of the experience since we are interested in the relation between *aesthetic emotions* and *pleasure* (though many subjects discussed what seems to be attentional properties of the stimulation). We also inquired into the *narratology* of chill-eliciting scenes. To do so, we asked by means of a survey 60 students from the same population (30 females; age range: 20–33; mean: 22.77) to describe a scene in a narrative (a film, a book, a play) that gave them aesthetic chills. In total, 23 descriptions were usable for this analysis (in the other cases, subjects did not remember, did not answer, or described scenes eliciting horror chills rather than aesthetic chills). The request was introduced with an open question: “Please describe as precisely as possible a scene in a film, a story or a play that gave you goose bumps (or chills, shivers, feeling of cold in your back, etc.).” We first analyzed the verbal reports paying attention to any redundancy that might be found in them. For the purpose of further experimentation, in what follows, we present these redundancies and the classes of situations eliciting chills using a general model to which researchers from all fields may easily refer (Polti, 1921). This model has the advantage of describing a wealth of scenes from different works of literature that may be used for further experimentations on aesthetic chills in the laboratory.

## Results

### Quantitative studies

Seven participants reported to have experienced chills in the course of the stimulation. In order to assess the dependency between the presence of chills during the experiment and the priming condition, we first draw the contingency table of these variables (Table 1). Chills were non-randomly distributed within the two priming groups as none of them were found in Group 3 (the incoherent prime).

**Table 1:** **Distribution of chill subjects within priming groups and mean pleasure**.

	**Chills (count)**	**No chills (count)**	**Pleasure**
Group1 (Pascal)	4	6	*M* = 7.2; *SD* = 1.5
Group 2 (Neutral)	3	7	*M* = 6.8; *SD* = 1.2
Group 3 (Chomsky)	0	10	*M* = 5.7; *SD* = 1.3

**Figure 2:**
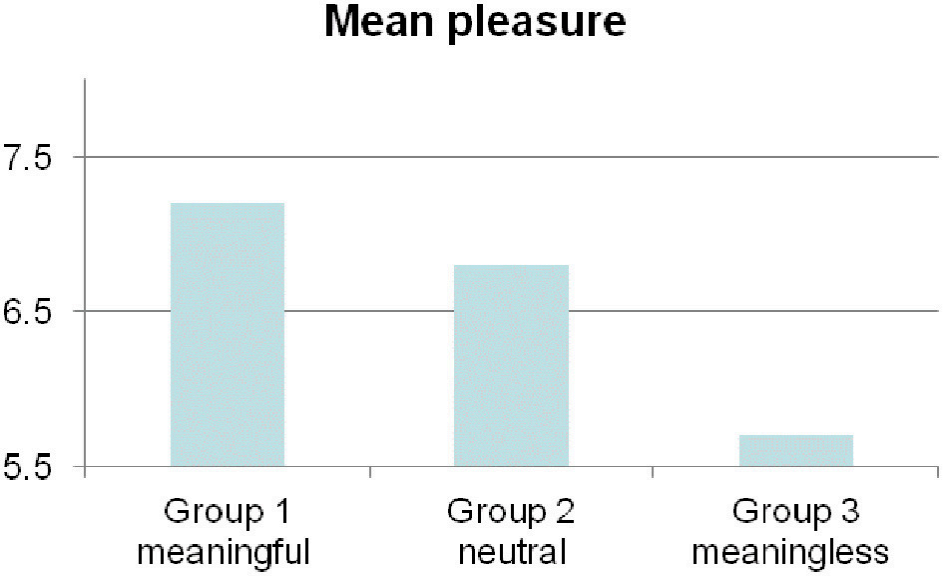
**Subjects primed with the Pascal sentence reported to have felt more pleasure than those primed with the Chomsky sentence**.

The data for each group are not inconsistent with a Gaussian distribution, all normality tests (Shapiro-Wilk) reported a *p* > 0.05. Since < 80% of the theoretical sample size is >5, we used an exact Fisher test to test the significance of these results (Alan, 1992). We observed a difference in chill responses between participants in Group 1 (*N*_chills_ = 4, *N*_group_ = 10) and participants in Group 3 (*N*_chills_ = 0, *N*_group_ = 10). A Fisher exact test indicated that these results are significant (*p* < 0.05). Hence, at any significance level, this test rejects the null hypothesis of independence between priming and chills occurrence in these groups. A Fisher exact test (*p* = 0.5) indicated that no significant difference in chill-response were found between participants in Group 1 (*N*_chills_ = 4) and participants in Group 2 (*N*_chills_ = 3). Likewise, no significant difference in chill-response were found between participants in Group 2 and participants in Group 3 (*p* = 0.105). We then proceeded to analyze the reports from the analog rating scale (Figure 2) and found a marginally significant difference in pleasure between the three priming conditions [*F*_(2, 27)_ = 3.175, *p* = 0.058], Group 1 scoring the highest (*M* = 7.2, *SD* = 1.55) and Group 3 the lowest (*M* = 5.7, *SD* = 1.34). To explore these results further we performed *post-hoc t*-tests by means of a Bonferroni correction. We found a significant difference [*t*_(18)_ = 2.318, *p* = 0.032] between Group 1 and 3.

### Qualitative studies

#### Narratology

This subsection is summarized within Table 2.

**Table 2:** **Chill-eliciting films and genre**.

**Film**	**Country of production**	**Year of production**	**Genre**
Saving Private Ryan	US	1998	drama
Titanic	US	1997	drama
Avatar	US	2009	adventure
Todo Sobre su Madre	Spain	1999	drama
Matrix	US	2003	science-fiction
12 Years a Slave	US	2013	drama
Panic Room	US	2002	drama
Good Will Hunting	US	1997	drama
Derek	UK	2012	drama
Helium	Denmark	2014	drama
Hunger Games	US	2012	adventure
Harry Potter	US	2011	adventure
Lion King	US	1994	adventure
The Notebook	US	2004	drama
Across the Universe	US	2007	drama
Whiplash	US	2014	drama
Dark Knight Rises	US	2012	thriller
The Notebook	US	2004	drama
Taken	US	2008	thriller
The Bridges of Madison County	US	1995	drama
Dead Poet Society	US	1989	drama
Interstellar	US	2014	adventure
The Others	US	2001	thriller
Green Mile	US	1999	drama
The Beach	US	2000	adventure

##### Properties of chill-eliciting scenes

More than 80% of the chill-eliciting scenes described by our subjects take place in the third act of a film and involve music (see Table 2). Two film genres are largely predominant: drama films and adventure films (IMDB's classification). Chill-eliciting scenes usually involve radical changes in the *relations* among characters; e.g., separation through death or reunion after strong and demanding efforts (here 62% of our scenes). These relations are usually of three kinds: (i) social relations, (ii) family relations, most notably parents-to-children relations, and (iii) couple relations. In case of a reunion scene, the amount of pleasure seems to be proportional to the quantity of effort necessary for the reunion (Schoeller and Perlovsky, 2015). Chills can also be enhanced by the presence of a strong change in the affective display of one of the characters (when empathy is a necessary condition to reduce narrative tension to a minimum).

Chills are also experienced when characters are no longer acting for their own sake but for a cause greater than themselves. As when a character is lying to others in order to protect them (i.e., hiding something fundamental for himself but harming for others) or when sacrificing themselves to something extraneous. That is, when the values driving a given action are stronger than the likelihood for this action being successful (which are usually null, as in sacrifice, heroism, bravery toward the impossible, etc.). Another important factor is the disclosure of the plot (58% of our sample), chill-eliciting scenes are usually a crucial node in the narrative which they belong to—i.e., the summary of the film would not be the same if the properties of these scenes were to be different (see the Discussion section for the implications of this fact).

##### Classes of situations eliciting chills

The 36 Dramatic Situations is a descriptive list created by Georges Polti (1921) to categorize every dramatic situation that might occur in a narrative. His work was influenced by Goethe and Carlo Gozzi. For each situations, Polti details the “indispensable elements” and provides extended explanations and examples. Examples of situations could be entire work of literature (e.g., Aeschylus' *Suppliants*), parts of a work (e.g., the third act of Voltaire's *Tancred*), historical examples (e.g., a historical episode of the crusades) or situations of ordinary life (e.g., claiming the body of a dead relative in the hospital). Here, we present the various situations found in our sample, provide the examples offered by Polti and add films quoted by participants.

- Deliverance (Polti's 2nd situation): involves an unfortunate, a threatener, a rescuer; in which an unexpected protector, of his own accord, comes suddenly to the rescue of the distressed and despairing. The first two subclasses of this situation are found in our sample of scenes:
  - Appearance of a rescuer to the condemned: Sophocles' “Andromeda”; the first act of “Lohengrin,” the third act of Voltaire's “Tancred”; the denouement of “Bluebeard.” In our sample: Panic Room (Fincher, 2002).
  - A parent replaced upon throne by his children: most notably “Aegeus” and “Peleus” by Sophocles; Euripides's “Antiope.” In our sample: The Lion King (Allers and Minkoff, 1994).
- Disaster (Polti's 6th situation): involves a vanquished power, a victorious enemy or a messenger. This situation is best defined by a great reversal of roles, the powerful are overthrown, the weak exalted. Most notably A. 2 a fatherland destroyed as in Byron's “Sardanapalus” and “The War of the Worlds” by Wells. In our sample: Hunger Games (Ross, 2012). Also, C. 1 (Ingratitude suffered), sacrifice unappreciated by those who have benefited by it, as in the “Timon of Athens” and “King Lear” of Shakespeare's; In our sample: Whiplash (Chazelle, 2014).
- Adventures (Polti's 9th situation): involves a bold leader, an object and an adversary. Two subclasses of this situation are noticeable in the descriptions provided by our subjects:
  - Preparations for war: in this subclass, the action stops before the denouement which it leaves to be imagined in the perspective of enthusiastic prediction. e.g., Aeschylus' “Nemea”; “The Council of the Argives” by Sophocles. In our sample: The Dark Knight Rises (Nolan, 2012), Avatar (Cameron, 2009).
  - Adventurous expedition: the usual exploits of the heroes of fairy tales. Euripides' “Theseus”; Sophocles' “Sinon.” the Labors of Hercules; the majority of Jules Verne's stories. In our sample: Interstellar (Nolan, 2014), The Beach (Boyle, 2000).
- Sacrifice (Polti's 20th and 21st situations). There are mainly two types of sacrifices quoted by our participants: self-sacrificing for an ideal which involves a hero, an ideal and the “creditor” or the person or thing sacrificed. Here, Polti notes that 'the field of conflict is no longer the visible world' (Polti, 1921, 67). It finds familiar instances in all martyrs. In fiction: “L'Œuvre” by Zola and “L'enfant du Temple” (de Pohles). In our sample: Saving Private Ryan (Spielberg, 1998). The second type of sacrifice is self-sacrifice for kindred which involves the hero, the Kinsman and the “creditor or the person or thing sacrificed.” Most notably C(2) Sacrifice for the happiness of one's child—e.g., “Le Réveil” (Hervieu, 1904); “La Fugitive” (Picard, 1911); in our sample: Titanic (Cameron, 1997).

#### Phenomenology of chill episodes

This subsection is summarized within Table 3.

**Table 3:** **Phenomenology of chill episodes**.

**Effect**	**Description**	**Quotes**
*Relaxation*: Psychologically, similarity (*L*) reaches a maximum. Physiologically, midbrain dopaminergic neurons reward the system.	Most subjects mention a strong relaxation and a calming effect. We note that some mention both curiosity and relaxation.	−Something calm and soothing that made me relax.
		−I was intrigued and calm at the same time.
		−Soothed and relaxed.
		−I felt far more relaxed, almost enchanted.
		−I was quiet relaxed.
		−It's very calming.
		−Peaceful and curious at once.
		−Relax and curious of what was going to happen.
		−I felt very relaxed.
		−I felt a sense of peace and quiet.
*Neutrality:* Psychologically, derivatives of *L* tend toward zero, thus the emotion is experienced as neutral.	In terms of emotional valence, it seems as chill episodes are neither positive nor negative.	−Neither a positive nor a negative moment, but a moment through which you don't belong to yourself anymore.
		−You can't divide the feeling on a dualistic level and say that it's either painful or pleasant.
*Psychological impact*: Categories at the top of the mental hierarchy were positively reinforced, highest models have been modified, the entire architecture of the cognitive system proved functional, something important was learned.	Subjects report a strong experience.	−It indicates that the movie has some sort of impact on me.
		−It was a very emotionally strong scene, and I like to get touched in such ways.
		−That always evokes a strong feeling of freedom in me.
		−The moment they started to dance seemed very emotional and dramatic.
		−It forces you to reconsider things.
*Arariskein*^a^: This effect is probably related to the activity of meaning-making.	Subjects describe some sort of concinnity effect, the harmonious adaptation of parts into a coherent meaningful whole.	−It made me focus on the bigger picture rather than the details of the images because of the atmosphere I was presented with.
		−The part where the small streams run together to form the river will send chills down my spine anytime [describing Smetana's *Má Vlast Moldau, Vltava*].
		−It was like watching a film where one person forces another to aggregate in order to produce a greater and more powerful whole.
*Empathy:* This effect could be due to the relation between cognition and social recognition, intelligence, and collective intelligence.	Subjects report an unusual strong empathy for the characters.	−I could say identification with the character.
		−I could feel the passion of the couple presented.
		−I felt carried away by the dancing couple.
		−When you connect with the character as a result in a film following them through their exploits, and a moment impacts upon you as much as them.
		−I kind of reacted the same way as the girl in the film.
		−Empathy for an emotional moment of the singer.
		−Could almost feel their sorrow myself.
*Authenticity*	Subjects talk about authenticity	−The end was disappointing, it looked like an ad and this destroyed the authenticity.
		−Disney movies and cartoons can give me goose bumps. But not romantic comedies. It has to seem authentic.
		−These films I guess ultimately make you question what is real in real life.
*Aesthetic criterion*	Subjects intentionally seek for this kind of moments in films, they sometimes use it as an aesthetic criterion.	−I would always seek it again.
		−I think it makes a good movie, song etc.
		−From having chills, I know that I like the film.
*Miscellaneous:* The manipulation of the category at the top of the mental hierarchy is mostly unconscious since difficult to manipulate and risky in terms of error as a modification of the entire world of the subject would follow but the emotion seems conscious.	Note that people do not seem to be reflecting so much about chills on a day-to-day basis, they usually do not know how to account for them and do not pay much attention to the event as such. They are surprised (and curious) when we bring the phenomenon to their attention.	−In post experimental interviews many subjects stated that they “never wondered about it,” “never thought of that before,” etc.

a This untranslatable term derives from the ancient Greek $\overset{´}{\alpha }\rho \alpha \rho \overset{´}{\iota }\sigma \kappa \omega$ (ararískō, “to join together”; PIE base ^*^ar- “to fit together.”) and can be more or less equated to the Gestalt effect, the cognitive system's ability to create forms to simplify raw perceptual data. For a general introduction to classical aesthetics (see Spitzer, 1963; Wittkower, 1960).

## Discussion

### Quantitative data

Experimentally, our working hypothesis is tentatively confirmed by the present results which seem to verify that (i) exposing subjects to an incoherent prime strongly decreases the likelihood of the subject experiencing chills during the subsequent exposition to a chill-eliciting stimulation and (ii) exposing subjects to a meaningful prime prior to the chill-eliciting stimulation makes the participant's experience more pleasurable. The fact that aesthetic chills are inhibited by incoherence demonstrates that the aesthetic experience is strongly dependent on meaning and our experimental protocol, as it seems replicable, therefore allows for a scientific study of both aesthetic emotions and meaning. We strongly encourage researchers to replicate this protocol in a different socio-cultural setting and with appropriate physiological measurements.

### Qualitative data

#### Narratology

In order to understand the structure of the scenes described by our subjects and their function in the narratives that they were extracted from, we should like to review some basic principles of narratology. Since Aristotle, it is generally accepted that narrative structures are a representation of human action (etymologically, drama means action). Human action is usually described as organized hierarchically (Barker and Wright, 1954; Miller et al., 1960). Each action is a step toward fulfillment of a plan, which is itself a contribution to a more comprehensive plan, and so on, until overriding plans corresponding to life goals are reached. Thus, ultimately, a successful story, just as a popular myth, should provide its audience with a better understanding of human action at the most general level (Polkinghorne, 1988). Most of the scenes described by our subjects are extracted from Hollywood drama narratives. These follow a specific pattern largely influenced by Aristotle's Poetics (Blocker, 2003) and a simple model describing this type of narrative structure is found in various manuals for storytellers widely used by contemporary scriptwriters (e.g., Campbell, 1949; Lavandier, 2005). The most basic elements of the structure are hero, goal, obstacle, and conflict. These elements are tied together by what we may refer to after Kreitler and Kreitler (1972) as *the tension principle*. The hero has a goal, he encounters obstacle, this creates tension (conflict) and the film ends when an answer to the dramatic question has been given (will the hero reach his goal?). Hollywood narratives can thus be described in terms of retention of information. The answer to the dramatic question is the information that the author retains. It is what the viewer cares about, *what motivates his curiosity*. As a consequence, if the audience suspects that the author does not hold such information, the spectatorial contract (i.e., “I am going to be told a story which end I shall know”) is broken, the tension principle ceases to apply, and the viewer has no good reason to wait until the end of the story. This, among many other things, is one of the reasons why one does not interrupt a theater play (he wants to know the end *as it will happen tonight* and if he interrupts the play, he will *never* know such end). This highly simplified explanation allows for a mathematical description. The hero (e.g., Bobby) has a goal *G* (e.g., he wants to become the best chess player in the world) but there exists an obstacle *O* (e.g., he does not play sufficiently well to beat his rival Garry). The amount of conflict, *c*, is inversely proportional to the likelihood of Bobby reaching his goal *G* given obstacle *O* (e.g., the less knowledge of chess, the greater the difficulty for Bobby to beat Garry at chess, the greater the amount of conflict). A story is the depiction of the process of Bobby acquiring enough knowledge to become the best chess player in the world. We may thus write the following structural equation:

$$\begin{array}{l} c\sim \;1∕P\left(G|O\right) \end{array}$$

Here, *c* is conflict and *P*(*G|O*) is the conditional probability of Bobby reaching his goal *G* given obstacle *O*. The lower the likelihood of success, the greater the narrative tension. This indeed is a highly simplified model. There are many characters in a given story and their goals are usually conflicting. This model is a metaphor, it serves to explore and explain processes not yet accessible to observation. We can use it to build testable predictions. From this highly simplified model, it seems possible to distinguish between at least two different types of stories. Stories where the conflict is a function of the hero wanting to achieve a general goal (e.g., money, fame, power) and stories where the conflict is a function of the hero wanting to avoid a general obstacle (e.g., sickness, catastrophe, misery). Stories involving conflicts of the first kind (internal conflict) have a different structure and trigger different emotions than stories involving conflicts of the second kind (external conflict). Narrative tension is directly proportional to conflict, and it is inversely proportional to the likelihood of the hero reaching his goal given obstacles.

All of the scenes described by our subjects involve the main character of the story that they were extracted from. The main character of a given story is generally defined as the hero that suffers the greatest amount of conflict (Lavandier, 2005). There exist mainly two types of heroes: (i) heroes who have tremendous goals (e.g., being the best chest player in the world) and (ii) heroes who have tremendous obstacles (e.g., surviving a natural catastrophe). Aristotle noted that there are mainly two types of tragedy both involving a great reversal in fortune. The first kind is a reversal from good to bad fortune (e.g., *Oedipus*) the second kind involves a reversal from bad to good fortune (e.g., *Eumenides*). According to him, the second is more pleasurable as it involves pity and fear and both are fundamental for *katharsis*, the *function* of tragedy. Most of the scenes described by our subjects fall under the second category (Good Will Hunting, Titanic, The Notebook, All About My Mother, 12 Years a Slave, Panic Room, etc.).

If the story is well-crafted (if the classical unities are respected, if the elements of the narrative are important, if the viewer cares about the story) narrative tension should trigger psychological tension (i.e., an emotional response). In the course of the story, tension increases and decreases. The reduction of tension causes pleasure (positive emotions) whereas the increase of tension causes displeasure (negative emotions). There are particular nods in the story (especially at the end, in the third act) where tension reaches a peak value. These nods constitute the structure of the story. They are usually contained in the simplest and shortest description of the story—i.e., its meaning (Chater, 1999; Dessalles, 2008). Popular blockbusters are often easily summarized in a few sentences and such is the case for most films in our sample. Aesthetic chills seem to occur when tension reaches a peak value. They occur in the last third of the plot when narrative tension reaches a global minimum. *Paradoxically*, if we consider the properties of the scenes they also seem to involve the resolution of an important conflict (thus the summary of the film would be different if the properties of these scenes were to be changed—it would not be the same story). The dynamic of our scenes therefore seems to involve a change from a great disequilibrium [P(G|O) ~ 0] to a state of equilibrium (c ~ 0). Consider for example the following scenes as described by our participants:

- *Panic Room:* there were three burglaries entering the house of a rich family: the family was in an safe room which was supposed to be for cases like this: the daughter was suffering from asthma and almost died at the end: at the end one of the burglaries shot the two other ones in order to save the daughter.
- *The Bridges of Madison County:* when Meryl Streep's looks back at the man she loves but can never be with. It made me feel sad and disappointed with the world.
- *The Imitation Game:* when they solve the Enigma code and find out that one's brother is on board of one of the ships being bombed by the Germans, but there's nothing they can do about it, because the enemy will get suspicious. They just all stand, looking at the map with tears in their eyes.
- The final scene of the film “*12 years a slave*” at the point when the main character (who is in silence for the majority of the film) is reunited with his family after his 12 year ordeal. He stands opposite to his wife and two children and their families. In this moment we see the life that he has missed. Very few words are said, initially it is a cagey and uncertain affair, the audience feels maybe too much has happened for any of the characters to be capable of understanding one another, but the tension ends with an outpouring of emotion and embracing. This is made more intense for the audience because we have seen how the main character has endured so much, but in this moment the weight of everything is released, he is softened and can once again feel.
- The scene in *Titanic* when the ship is about to sink and the camera focuses on a mother lying in bed with her children. At that point it is already clear that they will not being able to leave the ship before it sinks. Still, the mother is telling them a goodnight story.

The most remarkable thing to notice is that these scenes do not involve tension since the probable occurrence of a relevant event is not an undefined course of events. They do not provide clues for the viewer to predict future events. That is, the viewer's inferences find their conclusions in the properties constituting the scenes themselves (for further discussion refer to Schoeller and Perlovsky, 2015). There is no need to foresee what is going to happen next since the storyline has reached its point of equilibrium. The spectator is finally free to let go, the forces in the conflict field are balanced. At the narratological level, equilibria play an essential role. Chill-inducing scenes take place in the last third of the films; what Aristotle refers to as the third act. Freytag talks about *dénouement* (Freytag, 1897), the Russian formalist Vladimir Propp talks about “the hero's return” (Propp, 1968) and Todorov “a final equilibrium” (Todorov, 1969). By the end of most of the scenes from our sample, an answer to the dramatic question has been provided. This indeed corroborates the idea that disclosure of the plot seems to be a strong elicitor for aesthetic chills.

According to Hegel, tragedy emerges when a hero asserts a justified position, but in doing so simultaneously violates a contrary and similarly justified position (Hegel, 1975; Roche, 2006).

The original essence of tragedy consists then in the fact that within such a conflict each of the opposed sides, if taken by itself, has justification, while on the other hand each can establish the true and positive content of its own aim and character only by negating and damaging the equally justified power of the other (Hegel, 1975).

As noted by Roche, the nature of the tragic hero is therefore paradoxical as “greatness comes at the price of excluding what the situation demands” (the burglar should not save the daughter, Meryl Streep should not let her love go, the mother should not tell a story to her kids, the character in the *Imitation Game* should not sacrifice his brother—but, they are *forced* to do otherwise). In the words of Hegel, “it is the honor of these great characters to be culpable” and the tragic heroes embrace conflicting positions that are “equally justified” (Paolucci and Paulucci, 1962; Hegel, 1975; Roche, 2006). Here, it is possible to draw a parallel between Hollywood narratives and mythic structures in general. The structuralist theory of myth considers that the purpose of mythic structures is to provide a logical mode capable of overcoming contradictions (Lévi-Strauss, 1963, 226). The greater the contradiction resolved, the more powerful the myth.^1^ Studies in social cognition have shown that cognitive processes are heavily dependent on antinomies and polarization (e.g., see also Lupasco, 1947; Festinger, 1957; Lord et al., 1979, 1984) and so one might postulate that the universal success of Hollywood narratives and their general propensity to elicit chills might be due to their capability to help overcoming some of the most robust cognitive antinomies by presenting playful solutions to fundamental human conflicts. Following Festinger, we would thus propose that chill-eliciting scenes provide subjects with new (artificial) cognitive elements that maintain the *total dissonance of the system* at a rather low level (Festinger, 1957, 20) thus allowing the subject to divorce himself psychologically from the conflict (cf. Green and Brock, 2000). The basic background of Festinger's theory of cognitive consonance is coherent with the homeostatic model of cognition proposed in the introduction and consists in the notion that the human organism strives to establish internal harmony, consistency, or congruity among his opinions, attitudes, knowledge and values (Festinger, 1957, 260). However, depending on cultural or biological determinacies, there exist circumstances that naturally lead to fundamentally disharmonious, inconsistent and incongruent cognitions. These opposite pairs of cognitive elements (byproducts of knowledge-acquisition) remain infinitely resistant to change, this robustness being due to their irrevocability. The possibilities of change for such cognitions are almost nil since there is a clear and unequivocal reality corresponding to them (for further discussion see the discussion about the belief system of the Ifaluk in Festinger, 1957, 22). Empathy seems to play a crucial role in these scenes (e.g., in many of the descriptions, empathy is a necessary condition to reduce narrative tension to a minimum). In order to identify the kind of biological or cultural conflicts that may be responsible for such opposite pairs of cognition, we must turn our attention to the phenomenology of the experience.

#### Phenomenology

Our results indicate that viewers experience a strong relaxation during and after the chill episode and that chill-eliciting scenes have some sort of calming effect. An emotion is a psychological state or process that functions in the management of goals (Schoeller and Perlovsky, 2015 and references therein). It is positive when the goal is advanced, negative when the goal is impeded. As we already discussed, chill-eliciting scenes are reported in two film genres in particular: drama films and adventure films. Films from these categories involve complex situations of tensed conflict and thus are more prone to elicit chills since they are the center of a wide conflict fields (Grodal, 1997). Coherent with the narratological analysis, it is possible to draw a parallel here between these narratives and mythic structures in general. We proposed that the universal success of Hollywood narratives and their general propensity to elicit chills might be due to their capability to help overcoming some of the most robust cognitive antinomies by presenting *playful* solutions to fundamental human conflicts (Berlyne, 1957; Festinger, 1957). Chill-eliciting scenes provide subjects with *new cognitive elements* maintaining the total dissonance of the system at a rather low level thus allowing the subject to divorce himself psychologically from the conflict. Note, that this is coherent with the fact that piloerection in primates can be used as an indicator for internal conflict (Aureli and de Waal, 1977). This would also explain some other psychological effects resulting from engagement in narrative material (e.g., Gerrig, 1993; Green and Brock, 2000; Ankersmit, 2001; Cohen et al., 2015). The basic background of Festinger's theory of cognitive consonance consists in the notion that the human organism strives to establish internal harmony among his knowledge (Festinger, 1957). However, depending on cultural or biological determinacies, there exist circumstances that naturally lead to fundamentally disharmonious cognitions. These opposite pairs of cognition remain resistant to change since there is a clear and unequivocal reality corresponding to them. The strong and unusual empathy mentioned by our subjects leads us to propose that one of the fundamental human conflicts which chill-eliciting scenes might help resolve and overcome is the fact that humans survive by sharing goals but can never access to these goals directly (nobody will ever have directly access to your thoughts and conversely you will never have directly access to somebody else's thoughts). Another fundamental conflict that chill-eliciting stimuli might help overcome is the altruistic nature of human beings as opposed to the high degree of egoism presented by contemporary cultures (see for example Keltner, 2009; Fukui and Toyoshima, 2014 or Piff et al., 2015). The narratological results seem to suggest that they do so by displaying situations where empathy is necessary to reduce the narrative tension to a minimum. More empirical research would prove useful in improving our understanding of these processes.

## Conclusion

Scientific work devoted to the study of the apparently paradoxical function of aesthetic emotions in the cognitive system requires a combination of both strong theoretical arguments and detailed experimental results. Theoretically and in line with a philosophical tradition that has long associated aesthetic emotions to knowledge, we propose that aesthetic emotions correspond to a satisfaction/dissatisfaction of humans' knowledge instinct, the primary drive to acquire knowledge about the external and internal world and perceive events as meaningful (Hume, 1748; Kant, 1790/1987; Hamann, 2001/1758). This theory is coherent with the known biology of both aesthetic chills (Goldstein, 1980; Blood and Zatorre, 2001) and knowledge-acquisition (Kang et al., 2009; Jepma et al., 2012). Aesthetic chills were found to be significantly decreased or inhibited by injection of the synthetic opioid-antagonist naloxone (Goldstein, 1980) and laboratory studies in animal psychology provide extensive evidence that learning is influenced by opioid peptides (Martinez, 1981; Patterson et al., 1989). Furthermore, both knowledge-acquisition (Kang et al., 2009; Jepma et al., 2012) and aesthetic chills (Blood and Zatorre, 2001) activate striatal regions involved in reward processing and coding for vital parameters. However, besides the present experiment, no study has yet investigated the relation between aesthetic chills and knowledge-acquisition.

According to our working hypothesis, the aesthetic experience involving the content at the top of the cognitive hierarchy corresponds to an event when the mental representations encompassing *the widest range of reality* are positively reinforced by external cues, when the entire architecture of the cognitive system encounters supportive evidence that it is grounded on perennial and appropriate foundations. Hence, the subject is rewarded with pleasure and distributes his attentional resources accordingly. Such events, because they correspond to a change in similarity-knowledge, may vary in *degree* proportionally to the importance of the cognitive elements at play. The strength of emotion depends on the role played by these elements in the architectonics of the total system and the strength of the occurring response is a function of the amount of mismatch between bottom-up and top-down configurations (Schoeller and Perlovsky, 2015 and references therein). The highest levels of the hierarchy correspond to the most general and abstract models and their modification induces changes at all lower levels. Hence, such modifications are highly demanding in terms of resources, risky in terms of error and the content at the top of the hierarchy is highly difficult to manipulate consciously by the subject (Perlovsky, 2006, 2013a,b) which might explain why people do not reflect about chills on a day to day basis, why they are surprised when we bring this phenomenon to their attention. It resists change since an alteration of the subject's entire world would follow. These “highest” aesthetic experiences involve representations near the top of the mental hierarchy, which cognitive contents we experience mostly unconsciously as “meaning of life.” Acquiring knowledge about these representations even unconsciously invoke the emotion of the beautiful. Here, we are coming to understanding the nature of the beautiful not through a specific content, such as the visual features of a painting, but rather as what makes one's life meaningful.

This theory, though expressed in contemporary terminology, is coherent with a tradition in philosophy that has long associated aesthetic emotions to knowledge and has demonstrated major explanatory power in the field of musicology (Masataka and Perlovsky, 2012a,b, 2013; Perlovsky, 2012b,c, 2014b, 2015a; Perlovsky et al., 2013). In this study, we established a relation between aesthetic chills and the attribution of meaning. First, by showing that chills occur in a group exposed to meaningful prime, while an incoherent prime acts as a strong inhibitor for aesthetic chills, and second, by establishing that chill-inducing moments relate to meaning-making contents, content that is psychologically relevant for the subject. Though further experimentation should examine these relations in more detail, in current experiments our hypothesis has been tentatively confirmed, and we conclude from these results that aesthetic chills might correspond to a satisfaction of our participants' vital need for knowledge. A neural theory of how pleasure related to knowledge-acquisition and understanding arises from a network involving association cortex, dorsolateral prefrontal cortex, orbitofrontal cortex, striatum, opioids, and dopamine is outlined in Levine (2012). These detailed neural mechanisms of generalized mental representations are not yet accounted for in a simplified mathematical model of interacting emotions and cognition. An important aspect of these findings is that our experimental protocol allows for a scientific study of the physiology of aesthetic chills. Given that exposition to an incoherent prime strongly inhibits chills (as opposed to e.g., the sentence from Pascal), it is now possible for the experimenter to have two *groups of subjects*, both exposed to *the same stimulation* where subjects in one group experience chills while none of the other do so. Based on these results, we would like to suggest a series of propositions and hypotheses for further research:

- This study should be replicated in a different socio-cultural setting and with physiological measurements. As underlined throughout the article, our experimental protocol can be helpful to determine the physiology of the phenomenon. Given that incoherence seems to be such a strong inhibitor, an electrophysiological study on the problem of chills and the role of signals often related to the activity meaning-making (e.g., N400) in the activation/inhibition of chills might prove useful and shed some light on fundamental aspects of human nature (the relation between cognition/recognition, emotion/cognition, curiosity/pleasure/learning). Such a study, if added to the appropriate measurement tools might also clarify the problem of coherence and meaning. A physiological study could prove useful to verify the empirical findings of Goldstein (1980) and Blood and Zatorre (2001). It could also be of interest to determine whether ceremonial chills, musical chills, narrative chills, artistic chills, scientific chills, and religious chills correspond to the same biological event and if not what are the fundamental differences.
- Consistent with Berlyne's classic psychological theory of curiosity (Berlyne, 1960) and available data concerning aesthetic chills (Blood and Zatorre, 2001), biological studies of curiosity seem to demonstrate that curiosity activates brain regions sensitive to conflict and activates striatal regions involved in reward processing (Kang et al., 2009; Jepma et al., 2012). These studies also revealed a positive correlation between curiosity and memory retention; this might explain why participants mention a moment of psychological importance. Representations near the top of the hierarchy are mostly inaccessible to consciousness, still we suggest that the category “meaning of life” is at the top of the mental hierarchy and unifies the entire cognitive system. Priming the subject with this specific category may be a way to test this hypothesis. It should be done below the threshold of consciousness, as the subject will probably react antagonistically if exposed to the category consciously (given the very high degree of generality of the category and its unclear purpose, it is easier for the subject to dismiss the information rather than giving it its full attention). We hypothesize that the greater the attentional resources allocated by the subject on the primer, the stronger the emotion.
- We also advanced the hypothesis that aesthetic chills might be causally related to terrorist indoctrination. Participants in our studies repeatedly mentioned that the notion of sacrifice seems to play a causal role in the elicitation of chills and the classes of situations eliciting chills often correspond to those of propagandistic material at large (see e.g., Atran et al., 2014). This is coherent with previous results (e.g., Konečni et al., 2007, exp. 2; Haidt, 2000; Benedek and Kaernbach, 2011). Studies in general sociology show that when related to chills, the notion of sacrifice also plays a causal role in religious conversion (Inbody, 2015). We strongly encourage researchers specialized in security studies to pursue the analysis further by investigating the relation between humans' vital need for cognition, biological altruism, aesthetic chills and horror chills, and propaganda material.
- There does not exist any study on the ontogeny of chills (at what age do subjects start experiencing them? is there a relation between frequency of chills and age?) nor on the evolution of chills with cultures (how do chill-eliciting stimuli evolve over time? are there cultural differences?). Other studies have shown that chill-eliciting stimuli at large were found to cause an improvement of mood (Konečni et al., 2007; Benedek and Kaernbach, 2011). We also proposed that chills may help overcome some fundamental cognitive conflicts, pairs of cognition of equal resistance to change where the least resistant element may not be altered nor modified. Based on the subjective reports provided by the participants, we hypothesized two such conflicts: the vital need for other versus cognitive solitude and biological altruism versus cultural selfishness. Following Festinger's original intuition, we proposed that chill-eliciting stimuli might provide subjects with *new cognitive elements* maintaining the total dissonance of the cognitive system at a low level. It was hypothesized that Hollywood films do so by displaying playful solutions to fundamental human conflicts. Berlyne (1949) noted well that for knowledge of any given outcome to be rewarding, the event must be of some “interest” to the subject, it must be important, it must be meaningful—he equated this with the idea that strong habits or drives must be aroused. This is coherent with various accounts of the function of myths and narratives in human life: “unless the plot of the dream play represents a solution to a conflict in his spectators' life, the dream artist will have no audience at all, and hence cannot receive a social sanction for his play. On the other hand, if the plot of his play treats problems of great moment for his spectators, and treats them to their satisfaction in the sense that the spectators become clearer about the nature of their problems and of their solutions, then the theater may well become filled” (Zipf, 1949; see also, Lévi-Strauss, 1963; Laughlin and d'Aquili, 1974).
- It is interesting to note that some subjects relate their experience to immersion in a natural environment (e.g., beach, forest, mountain, etc.). The restorative value of nature as a vehicle to improve cognitive functioning has been the object of various studies in recent years (Kaplan, 2001; Cimprich and Ronis, 2003; Ottosson, 2007; Berto, 2014; Pearson and Craig, 2014). Exposure to restorative environments facilitates recovery from mental stress and fatigue (Berto, 2005). The attention restoration theory postulates that interacting with environments rich with inherently fascinating stimuli (e.g., sunsets) invoke involuntary attention moderately, allowing directed-attention mechanisms a chance to replenish (Kaplan, 2001). According to these studies, natural environments minimize the requirement for directed attention and therefore seem to have a restorative effects on cognitive functioning. After interacting with nature, subjects are able to perform better on tasks that depend on directed-attention abilities (Kaplan, 1995). Cognitive enhancement is also one of the known psychophysiological effects of music upon their listeners (e.g., Masataka and Perlovsky, 2012a,b; Perlovsky et al., 2013). More research on the relation between the aesthetic experience and nature interactions would be required before postulating any therapeutic outcomes (especially because of the location of the video used as a chill eliciting-stimulus for our experiments, not to mention the picture of the ocean we used to construct our baseline) but if such a relation were to be clearly established, this could open a promising future for the study of aesthetics and the therapeutic implications of aesthetic chills.
- It should be underlined that this study does not indicate whether the effects of some types of chill-eliciting scenes are more influenced by priming than others. In future research, it would appropriate to test the consequences of an incoherent prime on viewers exposed to different kinds of chill-eliciting scenes. The role of coherence in art appreciation has been well-studied (e.g., Zipf, 1949; Meyer, 1957; Wittkower, 1960; Arnheim, 1969, 1988). However, it is not yet completely clear why *incoherence* is such a strong inhibitor for aesthetic chills. In further research, it would be appropriate to study the effect of an incoherent prime on other universal emotions. Are subjects exposed to an incoherent prime prior to a stimulus likely to elicit sadness (e.g., a mother separated from her child) or fear (e.g., snakes, heights and other evolutionary relevant stimuli) less prone to react emotionally? if so, would the effect observed be as strong as the one observed in our experiments? Since, as underlined in many of our participants' reports, *harmony* seems to play a crucial role in the elicitation/non-elicitation of chills, it might also be useful to replicate this study on specific populations (e.g., musicians and mathematicians) and vary the prime in accordance (e.g., systemic integrity of a musical piece and elegant axiomatic of geometry). Such experimentations, if coupled with appropriate measurement tools of the physiology at play, could prove useful for determining if chills as elicited by audio-visual content pertains to the same class of biological phenomena as chills elicited by purely auditory or purely visual content. It could also shed some light on the psychobiological similarities and differences of chills as elicited by art, by science and by religion and on their respective roles in human societies.

## Author contributions

FS conceived and conducted the experiments, LP developed the theory, and both authors connected theory and experiments.

### Conflict of interest statement

The authors declare that the research was conducted in the absence of any commercial or financial relationships that could be construed as a potential conflict of interest.

## Footnotes

^1^We would like to underline that the classes of situation eliciting chills are closely related to the usual elements of propagandistic materials (sacrifice, martyrdom, preparation for war). More empirical evidence is provided by the only other study investigating narrative chills (Konečni et al., 2007). Given the current danger posed by such material, we encourage researchers in the field of propaganda and security studies to investigate chill-eliciting scenes and their psychological counterparts. Succinct though essential details regarding our database were provided, we encourage researchers to build their own and pursue the analysis further.
